# Blood Pressure Variability and End‐Stage Kidney Disease Among Individuals With Type 2 Diabetes: A Nationwide Cohort Study

**DOI:** 10.1111/1753-0407.70160

**Published:** 2025-10-23

**Authors:** Youn Huh, Hae‐Rim Kim, Hye Soon Park

**Affiliations:** ^1^ Department of Family Medicine, Uijeongbu Eulji Medical Center Eulji University Seongnam City Korea; ^2^ College of Natural Science, School of Statistics University of Seoul Seoul Korea; ^3^ Department of Family Medicine, Asan Medical Center University of Ulsan College of Medicine Seoul Korea

**Keywords:** blood pressure variability, end‐stage kidney disease, Korean, type 2 diabetes

## Abstract

**Background:**

Longitudinal evidence of the relationship between blood pressure (BP) variability and end‐stage kidney disease (ESKD) among individuals with type 2 diabetes is limited. Therefore, we evaluated the association between BP variability and ESKD in Korean adults with type 2 diabetes.

**Methods:**

The study utilized data from the Korean National Health Insurance Service database, comprising health checkups conducted between 2004 and 2015. We enrolled 36 421 adults aged ≥ 19 years with type 2 diabetes who underwent at least two health checkups and were followed up until the end of 2017. BP variability was measured using the coefficient of variation, standard deviation, and variability independent of the mean. Hazard ratios (HRs) and 95% confidence intervals (CIs) for ESKD were determined using multivariate Cox proportional hazards regression analysis.

**Results:**

During a median follow‐up of 8.05 years, 290 patients with ESKD were identified. The highest quartile of systolic or diastolic BP variability presented a higher risk of ESKD than did the lowest quartile of systolic or diastolic BP variability. The group with the highest systolic and diastolic BP variability had a 77% higher risk of ESKD than did those in the lowest three quartiles of both systolic and diastolic BP variability. These associations were present in younger individuals without comorbidities.

**Conclusions:**

Among individuals with type 2 diabetes, increased BP variability was associated with an increased risk of ESKD. These associations were similarly observed in younger individuals without comorbidities. Maintaining a consistent BP seems to be important to prevent progression to ESKD in individuals with type 2 diabetes.

## Graphical abstract

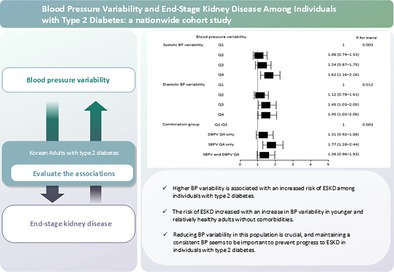

**Summary**

- Higher BP variability is associated with an increased risk of ESKD among individuals with type 2 diabetes.
- The risk of ESKD increased with an increase in BP variability in younger and relatively healthy adults without comorbidities.
- Reducing BP variability in this population is crucial, and maintaining a consistent BP seems to be important to prevent progress to ESKD in individuals with type 2 diabetes.

## 1 Introduction

End‐stage kidney disease (ESKD) is defined as an estimated glomerular filtration rate (eGFR) less than 15 mL/min/1.73 m^2^. The disease leads to potentially fatal complications without necessary interventions, such as hemodialysis, peritoneal dialysis (PD), and transplantation [1]. The social and personal burden of ESKD is high, and its prevalence is steadily increasing [2]. According to the United States Renal Data System ESKD database, the number of individuals with ESKD doubled from approximately 410 000 in 2001 to 810 000 in 2021 [2]. The same database demonstrated that the prevalence rates of ESKD also rose from 1644 per 100 000 to 2219 per 100 000 during the same period [2]. The prevalence of ESKD in Korea was 58 per 100 000 in 2007 and approximately tripled to 157 per 100 000 in 2015 [3].

Previous studies have shown that diabetes mellitus (DM) is an important risk factor for ESKD [4, 5]. DM affects kidney function, which could lead to the development and progression of chronic kidney disease (CKD) and result in ESKD [6]. In 2015, the mortality rate among patients with ESKD without DM was 2.6% in Korea, while that among patients with ESKD and DM was 4.3% [3]. Therefore, preventing ESKD among individuals with DM is crucial. Blood pressure (BP) is also significantly associated with CKD and ESKD [4, 7] and is one of the strongest cardiovascular risk factors [8]. Additionally, a study involving 2 865 157 US adults showed that high systolic BP variability (SBPV) was associated with an increased risk of ESKD [9]. Another study involving 48 587 Asians showed that high SBPV increased the risk of kidney function decline by 15% [10].

With regard to DM as a high‐risk factor for ESKD, a study involving 354 patients with type 2 diabetes showed that SBPV was associated with an increased risk of development of diabetic nephropathy [11]. However, this prior study involved small numbers of patients with type 2 diabetes and relied solely on SBPV, excluding diastolic BP variability (DBPV). The present study aimed to investigate the association of visit‐to‐visit variability in BP, including systolic BP, diastolic BP, and combinations of systolic and diastolic BP, with ESKD in a large cohort of Korean adults with type 2 diabetes, providing empirical data on this significant medical concern.

## 2 Methods

### 2.1 Data Source and Study Population

This study utilized nationwide cohort data obtained from the South Korean National Health Insurance Service (NHIS) database. This database contains information from a single universal insurer covering approximately 97% of South Koreans (approximately 50 million individuals). The NHIS covers national health checkups to be conducted at least every 2 years for all South Koreans aged ≥ 19 years, as well as for all employees. Therefore, the NHIS database includes comprehensive medical information regarding sociodemographic characteristics, lifestyle factors, health examinations, and medical diagnosis and treatment based on the International Classification of Diseases, 10th revision (ICD‐10) codes [12].

Among 148 517 participants diagnosed with type 2 diabetes between 2004 and 2011 (index year), 64 918 patients underwent at least two health examinations after type 2 diabetes diagnosis within 4 years. Among these patients, 64 911 who were aged ≥ 19 years were included. To avoid confounders due to pre‐existing diseases and minimize the possible effects of reverse causality, those who had a history of cancer and ESKD before the index year were excluded (*n* = 28 490). Ultimately, the study population comprised 36 421 individuals with type 2 diabetes.

### 2.2 Covariates

Anthropometric measurements, including height, weight, waist circumference, and BP (systolic and diastolic), were performed by healthcare professionals. Body mass index (BMI) was calculated as weight divided by height squared in meters (kg/m^2^). Obesity was defined as BMI ≥ 25.0 kg/m^2^ [13]. Blood samples were collected after overnight fasting to measure the concentrations of fasting plasma glucose, total cholesterol, and creatinine. Proteinuria was defined as a dipstick for protein ≥ +1.

Comorbidities were defined on the basis of a combination of health examination results and claims for medication prescriptions before the index date. Hypertension was defined as a systolic/diastolic BP of ≥ 140/90 mmHg or diagnosis with ICD‐10 codes I10–I13 or I15. Dyslipidemia was defined as a total cholesterol concentration of ≥ 240 mg/dL or a diagnosis with ICD‐10 code E78. CKD was defined as an eGFR of < 60 mL/min/1.73 m^2^ calculated using the modification of diet in renal disease equation.

Data on smoking status, alcohol consumption, and physical activity were collected using self‐reported questionnaires. Smoking status was divided into two categories: ever smokers and never smokers. Alcohol drinkers were defined as individuals who had consumed any form of alcohol at any point in their life. Regular exercise was defined as vigorous exercise for ≥ 3 days per week or moderate exercise for ≥ 5 days per week [14]. Low income was defined as falling in the lowest 20th percentile of income, using the NHI premium as a proxy for income level, and being eligible for medical aid.

### 2.3 Definitions of Blood Pressure Variability

The present study used the mean systolic and diastolic BP measured at each health checkup to calculate the standard deviations (SDs) in systolic and diastolic BP over various health checkups. Three indices of variability were used: SD, coefficient of variation calculated by dividing the SD by the mean BP [15], and variability independent of the mean (VIM). The VIM was measured as 100 × SD/mean *β*, where *β* is the regression coefficient [16]. The present study also calculated BP variability based on the number of BP measurements per participant, ranging from 3 to 5. SBPV or DBPV was divided into quartiles (systolic BP, Q1–Q4; diastolic BP, Q1–Q4). The highest variability of systolic (Q4) or diastolic BP (DQ4) was defined as the highest quartile for each parameter, and it was compared with that of the lower 3 quartiles (Q1–3) as the reference group.

### 2.4 Study Outcomes and Follow‐Up

The study population was followed up from baseline to the date of ESKD diagnosis or until December 31, 2019. The endpoint of the present study was newly diagnosed ESKD. ESKD was defined as a combination of ICD‐10 codes (N18‐19, Z49, Z94.0, and Z99.2) and a special code (V code) assigned to the initiation of renal replacement therapy (hemodialysis, V001; PD, V003) and kidney transplantation (V005) during hospitalization. The codes for treatment or medical expense claims included V005 for kidney transplantation, V001 for hemodialysis, and V003 for PD. We excluded individuals without a previous diagnosis of CKD who had a transplant or dialysis code on the same date as the acute renal failure code and those on continuous renal replacement therapy or acute PD.

### 2.5 Statistical Analyses

Baseline characteristics, stratified by BP variability, are presented as means ± SDs for continuous variables or numbers (percentages) for categorical variables. Continuous variables were compared using general linear analyses, and categorical variables were compared using the chi‐square test. The ESKD event rate was calculated by dividing the number of ESKD cases by 1000 person‐years.

We conducted multivariate Cox proportional hazards regression analyses to evaluate the association between BP variability and the risk of ESKD. The results are reported as hazard ratios (HRs) and 95% confidence intervals (CIs). Four models were used: Model 1, which was not adjusted for any variables; Model 2, adjusted for age, sex, BMI, smoking status, alcohol consumption, physical activity, and income; Model 3, adjusted for proteinuria and comorbidities (hypertension, dyslipidemia, and CKD); and Model 4, adjusted for mean BP (systolic BP for SBPV, diastolic BP for DBPV, and both systolic and diastolic BP for the combination group) in addition to the variables in Model 3. Subgroup analyses were conducted based on age, sex, comorbidities (hypertension, dyslipidemia, and CKD), and obesity. All statistical analyses were performed using SAS software (version 9.4; SAS Institute, Cary, NC, USA). Statistical significance was set at *p* < 0.05.

### 2.6 Ethics Approval and Consent to Participate

This study was approved by the Institutional Review Board of Uijeongbu Eulji Medical Center (No. UEMC 2023‐03‐001). The requirement for written informed consent was waived because all data utilized in the analysis were anonymous and non‐identifiable, and the need to obtain informed consent was waived by the Institutional Review Board of Uijeongbu Eulji Medical Center (No. UEMC 2023‐03‐001). We also performed in accordance with the Declaration of Helsinki.

## 3 Results

### 3.1 Baseline Characteristics of Study Participants

Table 1 presents the baseline characteristics of the study participants stratified by BP variability. The mean age of participants was 57.8 ± 10.0 years, with the highest mean age observed in the systolic BP VIM Q4 group. The proportion of men was 65.2%, that of ever‐smokers and drinkers was 40.6%, that of individuals who exercised regularly was 32.9%, and the prevalence of obesity was 56.4%. The mean BMI and FPG were 25.6 ± 3.4 mmHg and 131.2 ± 51.3 mmHg, respectively. The mean systolic and diastolic BP values were 130.0 ± 51.3 mmHg and 80.1 ± 10.3 mmHg, respectively. The prevalence of proteinuria was the highest among participants in the highest quartiles of SBPV and DBPV. Hypertension was least prevalent among participants in the lower quartiles of both SBPV and DBPV, whereas the mean eGFR was the highest among participants in the lower quartiles of both SBPV and DBPV.

**TABLE 1:** Baseline characteristics of study participants stratified by blood pressure variability.

	Blood pressure variability			SBPV and DBPV Q4	*p*
	Q1–Q3	DBPV Q4 only	SBPV Q4 only		
	(*N* = 22 629)	(*N* = 4452)	(*N* = 4686)	(*N* = 4654)	
Sex (men)	15 047 (66.5)	2847 (64.0)	2932 (62.6)	2924 (62.8)	< 0.001
Age (years)	57.3 ± 10.0	58.5 ± 10.2	59.2 ± 9.8	58.3 ± 9.7	< 0.001
Urban residence	4995 (22.1)	997 (22.4)	930 (19.9)	1011 (21.7)	0.006
Low income	570 (2.5)	111 (2.5)	134 (2.9)	118 (2.5)	0.587
Ever smoker	8988 (41.2)	1696 (39.5)	1738 (38.7)	1806 (40.3)	0.005
Drinker	9312 (41.2)	1812 (40.7)	1795 (38.3)	1862 (40.0)	0.003
Regular exerciser	7568 (33.4)	1479 (33.2)	1519 (32.4)	1427 (30.7)	0.003
Obesity	12 887 (57.0)	2521 (56.6)	2530 (54.0)	2609 (56.1)	0.003
Proteinuria	1601 (7.1)	329 (7.4)	313 (6.7)	376 (8.1)	0.042
Hypertension	8586 (37.9)	2138 (48.0)	1946 (41.5)	2188 (47.0)	< 0.001
Dyslipidemia	4800 (21.2)	1009 (22.7)	1045 (22.3)	1024 (22.0)	0.080
Chronic kidney disease	862 (2.4)	519 (2.3)	107 (2.4)	122 (2.6)	0.609
Height (cm)	162.3 ± 9.0	161.5 ± 9.1	161.0 ± 9.0	161.3 ± 9.1	< 0.001
Weight (kg)	67.8 ± 11.0	67.2 ± 11.1	66.0 ± 11.0	66.7 ± 11.2	< 0.001
BMI (kg/m^2^)	25.7 ± 3.4	25.7 ± 3.4	25.4 ± 3.3	25.6 ± 3.5	0.001
FPG (mg/mL)	131.4 ± 50.6	130.8 ± 50.8	129.9 ± 52.1	131.8 ± 54.1	0.630
Systolic BP (mmHg)	129.4 ± 13.5	131.8 ± 15.1	129.3 ± 19.6	131.9 ± 22.3	< 0.001
Diastolic BP (mmHg)	79.9 ± 8.6	80.9 ± 13.4	79.1 ± 9.2	81.4 ± 14.7	< 0.001
Total cholesterol (mg/mL)	204.6 ± 49.9	205.0 ± 43.8	203.6 ± 49.8	205.8 ± 68.5	0.479
eGFR (mL/min/1.73m^2^)	84.8 ± 27.6	82.7 ± 26.2	80.9 ± 26.2	83.1 ± 28.0	0.004
SBP variability, VIM	7.3 ± 3.9	8.8 ± 4.0	19.6 ± 4.4	22.9 ± 6.5	< 0.001
DBP variability, VIM	5.2 ± 2.8	13.7 ± 3.1	6.2 ± 2.8	15.6 ± 4.3	< 0.001

*Note:* Values are presented as means ± standard deviations or numbers (percentages).

Abbreviations: BMI, body mass index; BP, blood pressure; DBP, diastolic blood pressure; eGFR, estimated glomerular filtration rate; FPG, fasting plasma glucose; SBP, systolic blood pressure; VIM, variability independent of the mean.

### 3.2 Association of ESKD Incidence With BP Variability

During the median follow‐up of 8.05 (5.95–10.13) years, 290 (0.8%) new cases of ESKD developed. Table 2 shows the association between BP variability and the incidence of ESKD among individuals with type 2 diabetes. After adjusting for confounding variables, individuals in the highest quartile of SBPV had a 62% higher risk of ESKD than did those in the lowest quartile of SBPV (Model 4, HR 1.62, 95% CI: 1.16–2.26). Compared with the lowest quartile of DBPV, the risk of ESKD was highest in the highest (HR 1.45, 95% CI: 1.02–2.06) and third highest quartiles of DBPV (HR 1.45, 95% CI: 1.03–2.05). Individuals in SBPV Q4 only groups had a 77% higher risk of ESKD (HR 1.77, 95% CI: 1.28–2.44) than did those in the lowest three quartiles of both SBPV and DBPV.

**TABLE 2:** Hazard ratios and 95% confidence intervals for end‐stage renal disease stratified by quartiles of blood pressure variability among individuals with type 2 diabetes.

	Total (*n*)	Event (*n*)	Person‐years	Rate^a^	HR (95% CI)^b^			
					Model 1^c^	Model 2^d^	Model 3^e^	Model 4^f^
Systolic BP variability								
Q1	9104	58 (6.4)	71 848	0.81	1 (ref)	1 (ref)	1 (ref)	1 (ref)
Q2	8807	60 (6.8)	70 135	0.86	1.06 (0.74–1.52)	1.02 (0.71–1.47)	1.06 (0.73–1.53)	1.06 (0.74–1.53)
Q3	9170	75 (8.2)	73 196	1.02	1.26 (0.90–1.78)	1.19 (0.84–1.68)	1.22 (0.87–1.73)	1.24 (0.87–1.75)
Q4	9340	97 (10.4)	74 285	1.31	**1.61 (1.16–2.23)**	**1.58 (1.14–2.20)**	**1.62 (1.16–2.27)**	**1.62 (1.16–2.26)**
*P* for trend					0.002	0.004	0.003	0.003
Diastolic BP variability								
Q1	8979	56 (6.2)	70 891	0.79	1 (ref)	1 (ref)	1 (ref)	1 (ref)
Q2	9220	68 (7.4)	73 747	0.92	1.16 (0.82–1.66)	1.11 (0.77–1.59)	1.13 (0.79–1.63)	1.12 (0.78–1.61)
Q3	9116	81 (8.9)	72 629	1.12	**1.41 (1.00–1.98)**	1.39 (0.99–1.97)	**1.45 (1.02–2.05)**	**1.45 (1.03–2.05)**
Q4	9106	85 (9.3)	72 197	1.18	**1.49 (1.06–2.08)**	**1.47 (1.04–2.08)**	**1.45 (1.02–2.06)**	**1.45 (1.02–2.06)**
*P* for trend					0.010	0.011	0.013	0.012
Combination group								
Q1–Q3	22 629	155 (6.9)	179 952	0.86	1 (ref)	1 (ref)	1 (ref)	1 (ref)
DBPV Q4 only	4452	38 (8.5)	35 227	1.08	1.25 (0.88–1.79)	1.33 (0.93–1.91)	1.32 (0.92–1.88)	1.31 (0.92–1.88)
SBPV Q4 only	4686	50 (10.7)	37 315	1.34	**1.55 (1.13–2.13)**	**1.66 (1.20–2.28)**	**1.72 (1.25–2.38)**	**1.77 (1.28–2.44)**
SBPV and DBPV Q4	4654	47 (10.1)	36 970	1.27	**1.47 (1.06–2.04)**	**1.45 (1.04–2.04)**	1.39 (0.99–1.97)	1.36 (0.96–1.93)
*P* for trend					0.002	0.001	0.002	0.003

*Note:* Bolded values indicate significance at *p* < 0.05.

Abbreviations: BP, blood pressure; CI, confidence interval; HR, hazard ratio.

^a^ End‐stage renal disease per 1000 person‐years.

^b^ HRs (95% CIs) were calculated using multivariate Cox hazard regression analysis.

^c^ Model 1 was not adjusted for any variables.

^d^ Model 2 was adjusted for age, sex, body mass index, smoking status, alcohol consumption, physical activity, and income.

^e^ Model 3 was adjusted for age, sex, body mass index, smoking status, alcohol consumption, physical activity, income, hypertension, dyslipidemia, proteinuria, and chronic kidney disease.

^f^ Model 4 was adjusted for age, sex, body mass index, smoking status, alcohol consumption, physical activity, income, hypertension, dyslipidemia, proteinuria, chronic kidney disease, and mean blood pressure (systolic BP for systolic BP variability, diastolic BP for diastolic BP variability, and both systolic and diastolic BP for the combination group).

Table 3 presents the sensitivity analysis, excluding end‐stage renal disease within 2 years of follow‐up among individuals with type 2 diabetes. Individuals in the highest quartile of SBPV had a 2.3 times higher risk of ESKD than those in the lowest quartile of SBPV (Model 3, HR 2.29, 95% CI: 1.00–5.26). Compared with the lowest 3 quartiles of both SBPV and DBPV, the risk of ESKD was highest in both the SBPV and DBPV Q4 groups (HR 2.75, 95% CI: 1.17–6.46), followed by the SBPV Q4 group (HR 2.61, 95% CI: 1.11–6.14).

**TABLE 3:** Sensitivity analysis excluding end‐stage renal disease within 2 years of follow‐up among individuals with type 2 diabetes.

	Total (*n*)	Event (*n*)	Person‐years	Rate^a^	HR (95% CI)^b^			
					Model 1^c^	Model 2^d^	Model 3^e^	Model 4^f^
Systolic BP variability								
Q1	9104	8 (0.9)	18 113	0.44	1 (ref)	1 (ref)	1 (ref)	1 (ref)
Q2	8807	6 (0.7)	17 523	0.34	0.78 (0.27–2.23)	0.72 (0.25–2.08)	0.77 (0.27–2.21)	0.76 (0.27–2.16)
Q3	9170	10 (1.1)	18 232	0.55	1.24 (0.49–3.15)	1.10 (0.44–2.78)	1.14 (0.45–2.88)	1.19 (0.47–3.00)
Q4	9340	19 (2.0)	18 534	1.03	**2.32 (1.02–5.30)**	**2.30 (1.00–5.28)**	**2.29 (1.00–5.26)**	2.18 (0.92–5.14)
*P* for trend					0.027	0.034	0.037	0.052
Diastolic BP variability								
Q1	8979	7 (0.8)	17 842	0.39	1 (ref)	1 (ref)	1 (ref)	1 (ref)
Q2	9220	10 (1.1)	18 344	0.55	1.39 (0.53–3.65)	1.23 (0.46–3.29)	1.23 (0.46–3.33)	1.20 (0.44–3.25)
Q3	9116	8 (0.9)	18 132	0.44	1.12 (0.41–3.10)	1.04 (0.37–2.88)	1.06 (0.38–2.99)	1.02 (0.36–2.86)
Q4	9106	18 (2.0)	18 084	1.00	**2.54 (1.06–6.07)**	**2.43 (1.01–5.87)**	2.26 (0.92–5.52)	2.28 (0.93–5.64)
*P* for trend					0.049	0.063	0.080	0.086
Combination group								
Q1–Q3	22 629	17 (0.8)	45 020	0.38	1 (ref)	1 (ref)	1 (ref)	1 (ref)
DBPV Q4 only	4452	7 (1.6)	8848	0.79	2.10 (0.87–5.05)	2.29 (0.94–5.56)	2.11 (0.87–5.11)	2.23 (0.92–5.44)
SBPV Q4 only	4686	8 (1.7)	9298	0.86	2.28 (0.98–5.28)	**2.58 (1.11–5.99)**	**2.56 (1.10–5.95)**	**2.61 (1.11–6.14)**
SBPV and DBPV Q4	4654	11 (2.4)	9235	1.19	**3.15 (1.48–6.73)**	**3.25 (1.46–7.23)**	**2.96 (1.32–6.66)**	**2.75 (1.17–6.46)**
*P* for trend					0.001	< 0.001	0.002	0.004

*Note:* Bolded values indicate significance at *p* < 0.05.

Abbreviations: BP, blood pressure; CI, confidence interval; HR, hazard ratio.

^a^ End‐stage renal disease per 1000 person‐years.

^b^ HRs (95% CIs) were calculated using multivariate Cox hazard regression analysis.

^c^ Model 1 was not adjusted for any variables.

^d^ Model 2 was adjusted for age, sex, body mass index, smoking status, alcohol consumption, physical activity, and income.

^e^ Model 3 was adjusted for age, sex, body mass index, smoking status, alcohol consumption, physical activity, income, hypertension, dyslipidemia, proteinuria, and chronic kidney disease.

^f^ Model 4 was adjusted for age, sex, body mass index, smoking status, alcohol consumption, physical activity, income, hypertension, dyslipidemia, proteinuria, chronic kidney disease, and mean blood pressure (systolic BP for systolic BP variability, diastolic BP for diastolic BP variability, and both systolic and diastolic BP for the combination group).

### 3.3 Subgroup Analysis

**FIGURE 1:**
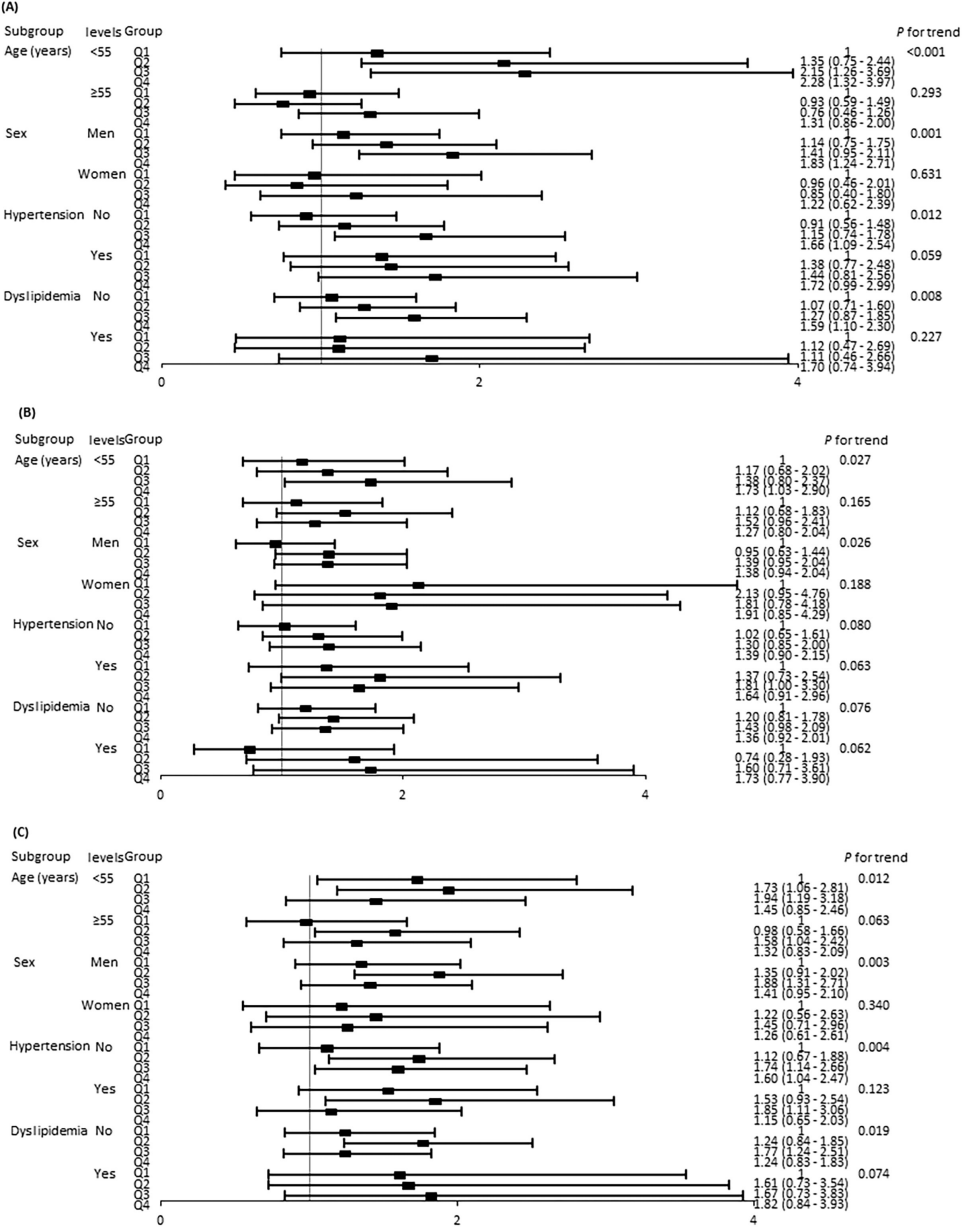
Subgroup analyses according to sex, age, comorbidities (hypertension and dyslipidemia), and obesity. (A) Systolic blood pressure variability. (B) Diastolic blood pressure variability. (C) Combination of systolic and diastolic blood pressure variability.

Figure 1 and Table 4 present stratified analyses according to age, sex, and comorbidities (hypertension, dyslipidemia, and CKD). Among young individuals, men, and those without comorbidities, individuals in the highest SBPV quartile had an increased risk of ESKD compared with those in the lowest SBPV quartile. Individuals in the highest DBPV quartile had an increased risk of ESKD compared with those in the lowest DBPV quartile among young individuals and individuals without CKD. Compared with individuals in the lowest three quartiles for both SBPV and DBPV, those in the highest quartiles for both SBPV and DBPV tended to have an increased risk of ESKD, except for women and individuals with dyslipidemia and CKD.

**TABLE 4:** Hazard ratios and 95% confidence intervals for end‐stage renal disease stratified by quartiles of blood pressure variability according to the presence of chronic kidney disease.

	Total (*n*)	Event (*n*)	Person‐years	Rate^a^	HR (95% CI)^b^			
					Model 1^c^	Model 2^d^	Model 3^e^	Model 4^f^
Chronic disease (−)								
Systolic BP variability								
Q1	8889	55 (6.2)	70 511	0.78	1 (ref)	1 (ref)	1 (ref)	1 (ref)
Q2	8591	56 (6.5)	68 814	0.81	1.04 (0.72–1.51)	1.00 (0.69–1.46)	1.00 (0.69–1.46)	1.04 (0.71–1.51)
Q3	8975	73 (8.1)	71 966	1.01	1.30 (0.91–1.84)	1.21 (0.85–1.72)	1.21 (0.85–1.73)	1.26 (0.88–1.80)
Q4	9104	93 (10.2)	72 782	1.28	**1.63 (1.17–2.28)**	**1.60 (1.14–2.25)**	**1.61 (1.14–2.26)**	**1.64 (1.16–2.31)**
*P* for trend					0.002	0.003	0.003	0.002
Diastolic BP variability								
Q1	8770	55 (6.3)	69 582	0.79	1 (ref)	1 (ref)	1 (ref)	1 (ref)
Q2	9014	64 (7.1)	72 461	0.88	1.11 (0.78–1.60)	1.06 (0.73–1.53)	1.06 (0.73–1.53)	1.08 (0.74–1.56)
Q3	8890	75 (8.4)	71 226	1.05	1.33 (0.94–1.88)	1.31 (0.92–1.87)	1.32 (0.93–1.87)	1.38 (0.97–1.96)
Q4	8885	83 (9.3)	70 803	1.17	**1.48 (1.05–2.08)**	**1.46 (1.03–2.07)**	**1.47 (1.04–2.09)**	**1.44 (1.01–2.05)**
*P* for trend					0.013	0.015	0.013	0.017
Combination group								
Q1–Q3	22 110	147 (6.7)	176 726	0.83	1 (ref)	1 (ref)	1 (ref)	1 (ref)
DBPV Q4 only	4345	37 (8.5)	34 565	1.07	1.29 (0.90–1.84)	1.37 (0.96–1.98)	1.38 (0.96–1.99)	1.34 (0.94–1.92)
SBPV Q4 only	4564	47 (10.3)	36 543	1.29	**1.54 (1.11–2.14)**	**1.65 (1.19–2.30)**	**1.66 (1.19–2.31)**	**1.77 (1.26–2.46)**
SBPV and DBPV Q4	4540	46 (10.1)	36 238	1.27	**1.52 (1.09–2.12)**	**1.50 (1.06–2.12)**	**1.51 (1.07–2.14)**	1.41 (0.99–2.00)
*P* for trend					< 0.001	0.001	< 0.001	0.002
Chronic disease (+)								
Systolic BP variability								
Q1	215	3 (14.0)	1337	2.24	1 (ref)	1 (ref)	1 (ref)	1 (ref)
Q2	216	4 (18.5)	1321	3.03	1.36 (0.30–6.14)	1.46 (0.32–6.60)	1.47 (0.32–6.68)	1.61 (0.35–7.42)
Q3	195	2 (10.3)	1231	1.63	0.73 (0.12–4.40)	0.83 (0.15–4.56)	0.83 (0.15–4.62)	0.83 (0.13–5.19)
Q4	236	4 (17.0)	1503	2.66	1.17 (0.26–5.15)	1.26 (0.29–5.47)	1.25 (0.29–5.51)	1.21 (0.27–5.39)
*P* for trend					0.984	0.919	0.923	0.994
Diastolic BP variability								
Q1	209	1 (4.8)	1309	0.76	1 (ref)	1 (ref)	1 (ref)	1 (ref)
Q2	206	4 (19.4)	1286	3.11	4.19 (0.47–37.74)	3.62 (0.38–34.71)	3.69 (0.38–35.48)	3.60 (0.36–36.34)
Q3	226	6 (26.6)	1402	4.28	5.71 (0.68–47.86)	5.17 (0.58–45.96)	5.42 (0.64–45.59)	5.40 (0.61–47.78)
Q4	221	2 (9.1)	1394	1.44	1.92 (0.17–21.47)	2.38 (0.20–28.09)	2.43 (0.21–28.87)	2.32 (0.16–34.31)
*P* for trend					0.482	0.306	0.286	0.356
Combination group								
Q1–Q3	519	8 (15.4)	3226	2.48	1 (ref)	1 (ref)	1 (ref)	1 (ref)
DBPV Q4 only	107	1 (9.4)	662	1.51	0.61 (0.08–4.92)	0.82 (0.09–7.29)	0.84 (0.09–7.60)	0.80 (0.07–8.64)
SBPV Q4 only	122	3 (24.6)	771	3.89	1.52 (0.40–5.69)	1.38 (0.38–4.97)	1.40 (0.38–5.19)	1.39 (0.39–4.95)
SBPV and DBPV Q4	114	1 (8.8)	732	1.37	0.55 (0.07–4.27)	0.72 (0.10–5.43)	0.70 (0.09–5.24)	0.60 (0.07–5.03)
*P* for trend					0.851	0.984	0.991	0.903

*Note:* Bolded values indicate significance at *p* < 0.05.

Abbreviations: BP, blood pressure; CI, confidence interval; HR, hazard ratio.

^a^ End‐Stage Renal Disease per 1000 person‐years.

^b^ HRs (95% CIs) were calculated using multivariate Cox hazard regression analysis.

^c^ Model 1 was not adjusted for any variables.

^d^ Model 2 was adjusted for age, sex, body mass index, smoking status, alcohol consumption, physical activity, and income.

^e^ Model 3 was adjusted for age, sex, body mass index, smoking status, alcohol consumption, physical activity, income, hypertension, dyslipidemia, proteinuria, and chronic kidney disease.

^f^ Model 4 was adjusted for age, sex, body mass index, smoking status, alcohol consumption, physical activity, income, hypertension, dyslipidemia, proteinuria, chronic kidney disease, and mean blood pressure (systolic BP for systolic BP variability, diastolic BP for diastolic BP variability, and both systolic and diastolic BP for the combination group).

## 4 Discussion

This study demonstrated an association between higher BP variability and the risk of ESKD using a nationwide, large‐scale cohort of individuals with type 2 diabetes. The highest SBPV was significantly associated with a 1.62‐fold increased risk of ESKD compared with the lowest SBPV. In addition, regarding DBPV, the risk of ESKD in individuals with higher DBPV was higher than that in those with the lowest DBPV. In the stratified analyses, these associations were significant in younger patients, men, and individuals without comorbidities.

Several studies have shown that greater BP variability increases the risk of ESKD in the general population. One study involving 2 864 157 American adults with a normal eGFR showed that higher SBPV was associated with an increased risk of ESKD; HRs for ESKD were the highest for individuals with the highest SBPV (HR, 10.59; 95% CI: 9.02–12.43), followed by those in the third (HR, 2.56; 95% CI: 2.16–3.03) and second quartiles (HR, 1.33; 95% CI: 1.10–1.60) [9]. Another study involving 165 191 Korean healthy adults aged ≥ 19 years demonstrated that the greater the BP variability in both systolic and diastolic BP, the higher the HRs of ESKD. The HRs for ESKD increased by 36% (HR 1.36, 95% CI: 1.30–1.42) and 34% (HR 1.34, 95% CI: 1.28–1.40) in the highest SBPV and DBPV groups, respectively, compared with the lowest SBPV and DBPV groups [17]. In addition, the HR for ESKD in both the SBPV and DBPV Q4 groups increased by 46% (HR 1.46, 95% CI: 1.40–1.53) compared with that in the lowest 3 quartiles for both SBPV and DBPV [17].

However, individuals with type 2 diabetes comprise a high‐risk group for ESKD and renal disease, as shown in a previous study where the prevalence of ESKD was 0.8% in individuals with type 2 diabetes and 0.2% in the general population [17]. The American Diabetes Association recommends that patients with type 2 diabetes should control their BP more strictly than should the general population [18]. Some studies have shown associations between BP variability and the incidence of ESKD and all‐cause mortality among individuals with type 2 diabetes [11, 19, 20]. One study involving 354 Japanese adults with type 2 diabetes demonstrated that SBPV was associated with an increased HR for the progression of albuminuria (HR 1.14, 95% CI: 1.01–1.30) [11]. Another study involving 2739 individuals with type 2 diabetes and nephropathy demonstrated that greater SBPV was associated with an increased risk of ESKD (HR 1.12, 95% CI: 1.02–1.22) and all‐cause mortality (HR 1.16, 95% CI: 1.04–1.29) [19]. One study found that the HRs for all‐cause mortality increased by 5% in SBPV (HR 1.05, 95% CI: 1.01–1.09) and 9% in DBPV (HR 1.09, 95% CI: 1.02–1.16), among 2161 Taiwanese individuals with type 2 diabetes [20]. However, the previous studies included a small number of patients with type 2 diabetes. We demonstrated that BP variability is an important risk factor for ESKD among individuals with type 2 diabetes using large‐scale population cohort data. The European Society of Hypertension guidelines for hypertension recommend maintaining consistent BP control for individuals undergoing hypertension treatment [21]. Similarly, maintaining consistent BP is crucial among individuals with type 2 diabetes.

Several possible mechanisms may underlie the impact of BP variability on the clinical course of patients with type 2 diabetes. First, increased BP variability might lead to increased renal vascular resistance, which is associated with eGFR and albuminuria [22, 23]. Higher BP variability induces impaired endothelial function by inhibiting nitric oxide production and increasing neointimal formation. Additionally, it contributes to atherogenesis [24]. Moreover, increased BP variability contributes to the progression of hypertension and kidney disease, which is caused by activation of the sympathetic nervous system [25]. Second, higher BP variability is associated with poor adherence to BP‐lowering medications and current smoking status [26]. It also increases the risk of albuminuria, endothelial dysfunction, carotid intima‐media thickness, and vascular rigidity [27]. These factors contribute to the association between increased BP variability and the progression of renal disease.

Even when stratified by age, sex, and comorbidities, the risk of ESKD was higher in the highest SBPV group among relatively healthy adults, younger adults, and individuals without comorbidities. DBPV showed this trend in younger individuals without CKD. In terms of BP variability combining systolic and diastolic BP, the risk of ESKD increased with SBPV in men and individuals without dyslipidemia or CKD, and this association was observed regardless of age or hypertension. In particular, variability in BP might be higher because of sympathetic activity in young adults [28]. Therefore, reducing BP variability might help reduce the risk of ESKD, even in healthy younger adults without comorbidities (hypertension, dyslipidemia, and CKD).

The present study has some limitations. First, although BP was measured according to the protocol provided by the NHIS during health checkups, it measured at the hospital may differ from home BP measurements. However, as most people tend to have their health checkups at the same hospital, no significant bias was introduced in investigating BP variability. Second, the retrospective design of the present study might have introduced reverse causality between BP variability and ESKD. Third, residual confounding factors, such as BP‐lowering medications [29] and the duration of type 2 diabetes [30], could have influenced the occurrence of ESKD among individuals with type 2 diabetes, although they were not accounted for in the NHIS database. Despite these limitations, our study demonstrated that variability in not only SBP but also DBP and a combination of the two was associated with the risk of ESKD in individuals with type 2 diabetes, unlike the findings in other studies [11, 19, 20]. In addition, we used a nationwide, large‐scale cohort of individuals with type 2 diabetes, the greatest risk factor for ESKD.

## 5 Conclusion

This study demonstrated that higher BP variability is associated with an increased risk of ESKD among individuals with type 2 diabetes. In addition, the study showed that the risk of ESKD increased with an increase in BP variability in younger and relatively healthy adults without comorbidities. Therefore, reducing BP variability in this population is crucial, and maintaining a consistent BP seems to be important to prevent progression to ESKD in individuals with type 2 diabetes.

## Author Contributions

**Youn Huh:** conceptualization, methodology, investigation, formal analysis, writing – original draft, writing – reviewing and editing. **Hae‐Rim Kim:** conceptualization, investigation, formal analysis, writing – reviewing and editing. **Hye Soon Park:** conceptualization, methodology, investigation, formal analysis, writing – original draft, writing – reviewing and editing.

## Ethics Statement

This study was approved by the Institutional Review Board of Uijeongbu Eulji Medical Center (No. UEMC 2023‐03‐001). The requirement for written informed consent was waived because all data utilized in the analysis were anonymous and non‐identifiable, and the need to obtain informed consent was waived by the Institutional Review Board of Uijeongbu Eulji Medical Center (No. UEMC 2023‐03‐001). We also performed in accordance with the Declaration of Helsinki.

## Conflicts of Interest

The authors declare no conflicts of interest.

## Data Availability

The data that support the findings of this study are available from the South Korean National Health Insurance Service, but restrictions apply to the availability of these data, which were used under license for the current study and so are not publicly available. Data are, however, available from the authors upon reasonable request and with permission of the South Korean National Health Insurance Service if it is the corresponding author.
